# Supplementary figures and images for: Clinical Characteristics and Predictors of In-Hospital Mortality among Older Patients with Acute Heart Failure
Source: J Clin Med. 2022 Jan 15;11(2):439. doi: 10.3390/jcm11020439 (PMC8781633; doi:10.3390/jcm11020439)

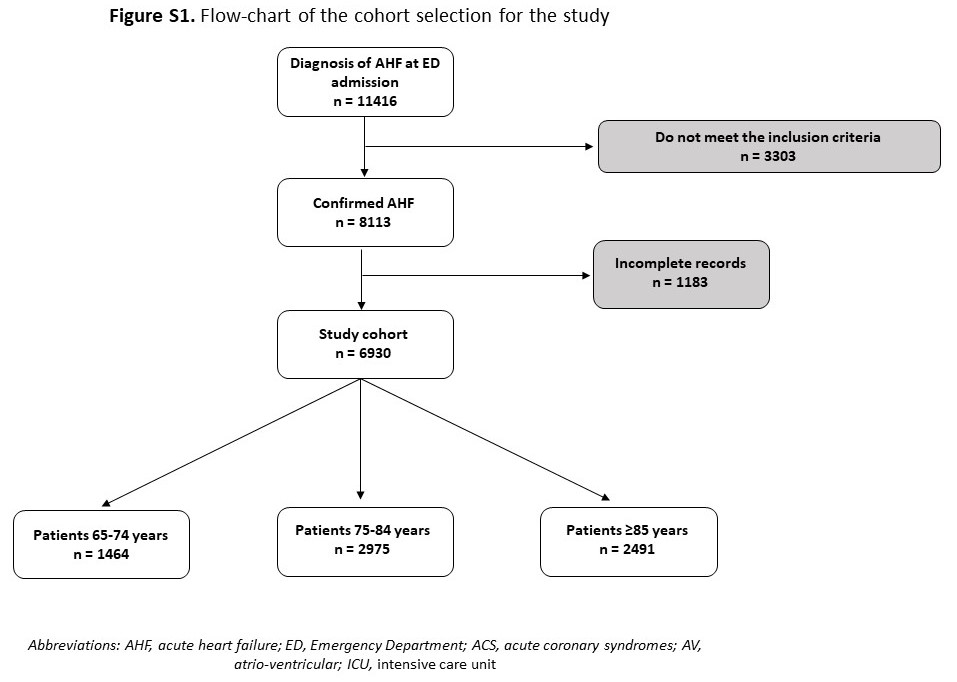

Supplement: Supplementary file 1 [file jcm-11-00439-s001.zip › jcm-1518792-supplementary.jpg]
